# Comparative Analysis of Growth Performance, Morphological Development, and Physiological Condition in Three Romanian *Cyprinus carpio* Varieties and Koi: Implications for Aquaculture

**DOI:** 10.3390/life14111471

**Published:** 2024-11-12

**Authors:** Dana-Andreea Șerban, Cristian-Alin Barbacariu, Marian Burducea, Mihaela Ivancia, Șteofil Creangă

**Affiliations:** 1 Research and Development Station for Aquaculture and Aquatic Ecology, “Alexandru Ioan Cuza” University, Carol I, 20A, 700505 Iasi, Romania; alin.barbacariu@uaic.ro (C.-A.B.); marian.burducea@uaic.ro (M.B.); 2 Faculty of Food and Animal Sciences, University of Life Sciences “Ion Ionescu de la Brad” Iaşi, Mihail Sadoveanu Alley 6-8, 700490 Iasi, Romania; mihaela.ivancia@iuls.ro (M.I.); steofil.creanga@iuls.ro (Ș.C.)

**Keywords:** Romanian carp varieties, morphological development, Fulton’s condition factor, ecological adaptation, ontogenic stages, genetic diversity

## Abstract

This study investigates the influence of internal factors on growth dynamics in four *Cyprinus carpio* varieties, three Romanian strains (Frăsinet, Ineu, and Podu Iloaiei) and the Koi variety. Fish were measured for total length, maximum height, and weight at four ontogenic stages, namely 7 days post-hatch, 3 months (0^+^), 18 months old (1^+^), and 36 months (2^+^). Weight Gain (WG), Specific Growth Rate (SGR), Relative Growth Rate (RGR), Fulton’s condition factor, and the profile index were calculated and analyzed. Results revealed significant intervariety differences in growth performance and physiological condition across life stages. At the 2^+^ stage, Podu Iloaiei exhibited the highest WG (849.73 ± 4.09 g), while Koi showed the lowest (403.99 ± 14.21 g). Koi demonstrated a unique growth pattern, with the highest SGR (0.18 ± 0.00% day^−1^) and RGR (0.98 ± 0.05 g day^−1^) at the 2^+^ stage. Fulton’s condition factor varied markedly, with Frăsinet showing the highest value at 7 days post-hatch (149.57 ± 17.485) and Koi the lowest at the 1^+^ stage (0.63 ± 0.011). The profile index decreased with age in all varieties, with Podu Iloaiei showing the most dramatic change from 4.22 ± 0.149 at 7 days to 2.18 ± 0.004 at 2^+^. These findings highlight the complex interplay between genetic and developmental factors in carp varieties, offering new insights for tailored breeding programs and aquaculture practices.

## 1. Introduction

The growth dynamics of fish have long been a subject of interest in ichthyology, aquaculture, and fishery management [1]. Understanding the internal factors influencing fish growth is important for optimizing aquaculture practices, managing wild fish populations, and predicting ecosystem responses to environmental changes [2,3].

Aquaculture has experienced a significant rise in recent years, becoming an increasingly important source of food production globally [4,5]. As the demand for aquaculture products continues to grow, there is a pressing need to enhance production efficiency and sustainability [6,7]. To achieve these goals, a deeper understanding of the internal factors influencing fish growth and development is essential. While external factors such as temperature, food availability, and water quality have been extensively studied [8], the internal regulatory processes driving growth variations among individuals and species remain less understood [9].

Fish growth is a multifaceted process influenced by both external environmental factors and internal physiological mechanisms [10]. Recent advances in molecular biology and endocrinology have shed new light on the intricate network of internal factors governing fish growth [11,12]. The growth hormone (GH) insulin-like growth factor-I (IGF-I) axis plays a central role in regulating fish growth [13]. The exact mechanisms by which this axis interacts with other endocrine systems, such as the thyroid and reproductive hormones, are still debated [14,15]. The relative importance of the GH-IGF-I axis versus local tissue-specific growth factors remains controversial [16].

Genetic factors also significantly contribute to growth variability in fish [17]. Quantitative trait loci (QTL) associated with growth-related traits have been identified in various species [18], but the functional roles of many growth-related genes and their interactions have yet to be fully elucidated [19]. The emerging field of epigenetics has further complicated our understanding of growth regulation, suggesting that environmental factors can influence gene expression patterns across generations [20].

Metabolic processes, including protein turnover, lipid metabolism, and energy allocation, are integral to fish growth [21]. The molecular mechanisms underlying metabolic efficiency and their relationship to growth performance are not fully understood [22]. The impact of gut microbiota on fish metabolism and growth has recently gained attention, opening new avenues for research [23].

This study aims to elucidate the complex interplay of endogenous factors that regulate fish growth by investigating four varieties of *Cyprinus carpio* (Frăsinet, Ineu, Podu Iloaiei, and Koi) at four different ontogenic stages. By examining these varieties across multiple life stages, we seek to shed light on how internal factors influence carp growth, providing valuable insights for aquaculture practices and breeding programs. This research contributes to a more comprehensive understanding of the endogenous mechanisms driving fish growth, potentially leading to improved aquaculture practices and more accurate models for fishery management. The findings may have significant implications for selective breeding programs, feed formulation strategies, and the development of novel growth-enhancing techniques in aquaculture.

## 2. Materials and Methods

### 2.1. Fish Samples and Rearing Conditions

This study examined 4 varieties of *Cyprinus carpio*, namely Frăsinet, Ineu, Podu Iloaiei, and Koi. The varieties were reared in separate earthen ponds within their respective facilities, reflecting standard commercial aquaculture practices. The Frăsinet variety was maintained at a private commercial farm in Valea Morii-Secuieni Neamț, Romania, where the pure race had been cultivated for over 10 years under consistent management practices in a series of three interconnected earthen ponds (each 0.5 ha, average depth 1.5 m). The other three varieties (Ineu, Podu Iloaiei, and Koi) were reared at the Research and Development Station for Aquaculture and Aquatic Ecology (SCDAEA) of “Alexandru Ioan Cuza” University in Iași, Romania, in separate but identical earthen ponds (0.4 ha each, average depth 1.5 m).

The pond system at each facility was equipped with independent water supply and drainage systems, allowing for individual pond management while maintaining consistent conditions across all experimental units. To account for potential pond effects, environmental parameters were monitored daily at standardized points in each pond (inlet, center, and outlet). At the SCDAEA, the three varieties were reared in adjacent ponds sharing the same water source and water management system to minimize environmental variation. Water exchange rates were standardized at 10% daily across all ponds, and each pond was equipped with aerators to maintain dissolved oxygen levels and prevent stratification. Bottom sediment quality and natural productivity were monitored monthly to ensure comparable growing conditions across all experimental units.

Fish were reared in outdoor earthen ponds under natural photoperiod and local climatic conditions. During the larval stage (first 7 days post-hatch), fish relied on natural pond productivity, primarily consuming zooplankton and other naturally occurring organisms [24]. From day 8 to 21, feeding transitioned to a combination of natural food and commercial micropowdered feed (60% protein, 12% lipids). From day 22 to the fingerling stage (0^+^), fish received commercial pelleted feed (35% protein, 8% lipids) at 5% of body weight daily, divided into three feedings. For the 1^+^ stage, the feeding rate was adjusted to 3% of body weight with commercial grow-out feed (30% protein, 7% lipids), provided twice daily, while 2^+^ stage fish were fed once daily at 3% body weight [24].

Environmental parameters were monitored daily. In the growth period, the temperature was within 20–26 °C, the established optimal range for common carp growth [24]. Dissolved oxygen was above 5 mg/L, pH between 6.5 and 8.5 [25], and total ammonia nitrogen below 0.5 mg/L [26]. These parameters align with established protocols for successful carp breeding programs [27].

### 2.2. Sampling, Equipment, and Measurements

The study monitored the following four critical ontogenic stages in carp development:

7 days post-hatch (larval stage): initial measurements marking the completion of yolk sac absorption and transition to exogenous feeding.

0^+^ (summer fingerlings): approximately 3 months old, representing completion of first intensive growth period.

1^+^: approximately 15 months old (two summers, one winter), capturing post-winter recovery and second summer growth.

2^+^: approximately 27 months old (three summers, two winters), representing market-size fish.

This classification aligns with standard aquaculture practices and reflects the seasonal growth patterns of *Cyprinus carpio*, where significant growth occurs during warmer months [24]. At each developmental stage, 30 individuals per variety were randomly selected for measurements (*n* = 120 per stage, total *N* = 480). To minimize handling stress, fish were anesthetized using MS-222 (100 mg/L, Sigma-Aldrich, St. Louis, MO, USA) following standard protocols [28].

The following morphometric parameters were measured using calibrated equipment:

Total length (TL): measured from the tip of the snout to the end of the caudal fin using an ichthyometer.
Maximum height (MH): measured at the deepest part of the body using the same ichthyometer.
Weight (W): determined using a calibrated electronic balance (Kern EMB 6000-1, precision 0.01 g).

For consistency, all measurements were performed by the same trained personnel. Fish were measured after 12 h of fasting to ensure emptied digestive tracts. For each ontogenic stage, initial weights were recorded at the beginning of the stage (corresponding to the final weight of the previous stage, except for 7 days post-hatch measurements). Final weights were recorded at stage completion, serving as initial weights for subsequent stages.

The following environmental parameters were monitored daily using calibrated equipment:

Temperature and dissolved oxygen: HQ30d flexiparameter (precision ±0.1 °C, ±0.1 mg/L).
pH: HQ11D waterproof meter (precision ±0.01 pH).
Total ammonia nitrogen: HI801 IRIS VISIBLE spectrophotometer (precision ±0.01 mg/L).

All equipment were calibrated monthly according to manufacturer specifications. Temperature and pH were taken at three standardized points in each pond at 8:00 AM and 2:00 PM daily, and total ammonia nitrogen was taken weekly.

Mortality was monitored daily throughout the study period. A stage-specific survival analysis revealed differential patterns, namely that larval and post-larval stages (0–21 days post-hatch) exhibited 28 ± 1.69% survival, while the fingerling stage (22 days to 0^+^) showed improved survival rates of 71 ± 2.62%. Survival continued to increase through grow-out phases, with the 1^+^ stage maintaining 87 ± 1.86% survival and final survival rates at the 2^+^ stage reaching 96 ± 0.68%. No statistically significant differences in mortality patterns were observed between varieties (*p* > 0.05), these values being normal for this species.

### 2.3. Growth Period Significance

The emphasis on summer growth periods in this study reflects the natural growth cycle of *Cyprinus carpio*. During summer months (water temperature 20–26 °C), carp exhibit maximum growth potential due to optimal metabolic rates and feeding activity [25]. Conversely, growth significantly slows during winter months when temperatures drop below 15 °C, making summer growth important for overall development [29]. This seasonal pattern is fundamental in temperate climates like Romania, where distinct growing seasons influence aquaculture production cycles.

### 2.4. Growth Performance Indices

The following indices were calculated to assess growth performance:

Weight Gain (WG) = Final Weight − Initial Weight
Specific Growth Rate (SGR) = [(ln Final Weight − ln Initial Weight)/time (days)] × 100
Relative Growth Rate (RGR) = [(Final Weight − Initial Weight)/Initial Weight] × 100
Fulton’s condition factor (K) = (Weight/Total Length^3^) × 100
Profile index = Maximum Height/Total Length

### 2.5. Statistical Analysis

All statistical analyses were performed using IBM SPSS Statistics version 26.0 (IBM Corp., Armonk, NY, USA). Data were first tested for normality using the Shapiro–Wilk test and for homogeneity of variances using Levene’s test. A one-way ANOVA was used to compare the growth indices among the four carp varieties at each life stage. When significant differences were found, post hoc Tukey’s HSD tests were conducted to determine which groups differed significantly.

## 3. Results

### 3.1. Growth Performance

#### 3.1.1. Weight Gain (WG)

At the 0^+^ stage, Frăsinet and Podu Iloaiei varieties showed significantly higher WG (37.55 ± 0.15 g and 37.37 ± 0.14 g, respectively) compared to Koi (36.75 ± 0.08 g) and Ineu (36.27 ± 0.11 g) varieties (F = 12.45, *p* < 0.001) (Table 1).

For the 1^+^ stage, Podu Iloaiei exhibited the highest WG (583.65 ± 1.68 g), followed by Ineu (560.79 ± 2.00 g), Frăsinet (471.13 ± 1.78 g), and Koi (77.73 ± 2.10 g). All differences were statistically significant (F = 24.67, *p* < 0.001) (Table 1).

At the 2^+^ stage, Podu Iloaiei, Ineu, and Frăsinet showed similar WG values (849.73 ± 4.09 g, 846.25 ± 5.17 g, and 834.45 ± 5.28 g, respectively) with no significant differences among them. Koi exhibited significantly lower WG (403.99 ± 14.21 g) compared to the other varieties (F = 18.92, *p* < 0.001) (Table 1).

#### 3.1.2. Specific Growth Rate (SGR)

For the 0^+^ stage, Podu Iloaiei, Koi, and Ineu showed significantly higher SGR values (2.73 ± 0.04, 2.70 ± 0.09, and 2.57 ± 0.07% day^−1^, respectively) compared to Frăsinet (2.20 ± 0.05% day^−1^) (F = 15.32, *p* < 0.001) (Table 2).

At the 1^+^ stage, Podu Iloaiei and Ineu exhibited the highest SGRs (0.33 ± 0.00% day^−1^ for both), followed by Frăsinet (0.31 ± 0.00% day^−1^) and Koi (0.13 ± 0.00% day^−1^). All differences were statistically significant (F = 21.84, *p* < 0.001) (Table 2).

For the 2^+^ stage, Koi showed the highest SGR (0.18 ± 0.00% day^−1^), followed by Frăsinet (0.12 ± 0.00% day^−1^), while Ineu and Podu Iloaiei had the lowest values (0.10 ± 0.00% day^−1^ for both) (F = 19.76, *p* < 0.001) (Table 2).

#### 3.1.3. Relative Growth Rate (RGR)

At the 0^+^ stage, Koi exhibited the highest RGR (508.59 ± 141.95 g day^−1^), which was significantly different from Frăsinet (119.98 ± 21.13 g day^−1^) (F = 3.27, *p* = 0.045). Ineu and Podu Iloaiei showed intermediate values (351.31 ± 122.65 and 322.27 ± 33.76 g day^−1^, respectively), with no significant differences from the other varieties (Table 3).

For the 1^+^ stage, Podu Iloaiei and Ineu showed the highest RGRs (4.26 ± 0.02 and 4.21 ± 0.02 g day^−1^, respectively), followed by Frăsinet (3.40 ± 0.02 g day^−1^) and Koi (0.58 ± 0.02 g day^−1^). All differences were statistically significant (F = 25.91, *p* < 0.001) (Table 3).

At the 2^+^ stage, Koi exhibited the highest RGR (0.98 ± 0.05 g day^−1^), which was significantly different from all other varieties (F = 17.83, *p* < 0.001). Frăsinet, Ineu, and Podu Iloaiei showed lower RGR values (0.45 ± 0.00, 0.39 ± 0.00, and 0.38 ± 0.00 g day^−1^, respectively), with no significant differences among them (Table 3).

### 3.2. Profile Index

At 7 days post-hatch, significant differences were observed among all varieties (F = 28.64, *p* < 0.001). Podu Iloaiei exhibited the highest profile index (4.22 ± 0.149), followed by Koi (3.69 ± 0.101), Frăsinet (3.06 ± 0.07), and Ineu (2.90 ± 0.07) (Table 4).

For the 0^+^ stage, Koi showed the highest profile index (2.93 ± 0.023), which was significantly different from all other varieties (F = 19.82, *p* < 0.001). Frăsinet had the second-highest value (2.57 ± 0.006), while Podu Iloaiei and Ineu showed lower similar values (2.45 ± 0.009 and 2.44 ± 0.009, respectively), with no significant difference between them.

At the 1^+^ stage, Koi maintained the highest profile index (2.48 ± 0.005), followed by Frăsinet (2.40 ± 0.005). Podu Iloaiei and Ineu showed lower similar values (2.18 ± 0.008 and 2.17 ± 0.005, respectively). All differences were statistically significant (F = 22.15, *p* < 0.001), except between Podu Iloaiei and Ineu.

For the 2^+^ stage, Frăsinet exhibited the highest profile index (2.43 ± 0.005), which was significantly different from all other varieties (F = 16.93, *p* < 0.001). Podu Iloaiei, Koi, and Ineu showed lower values (2.18 ± 0.004, 2.17 ± 0.005, and 2.16 ± 0.004, respectively), with no significant differences among them.

### 3.3. Fulton’s Condition Factor

Fulton’s condition factor was analyzed for the four *Cyprinus carpio* varieties at four different life stages, namely 7 days post-hatch, summer fingerlings (0^+^), one year and summer old (1^+^), and two years and summer old (2^+^).

**Figure 1:**
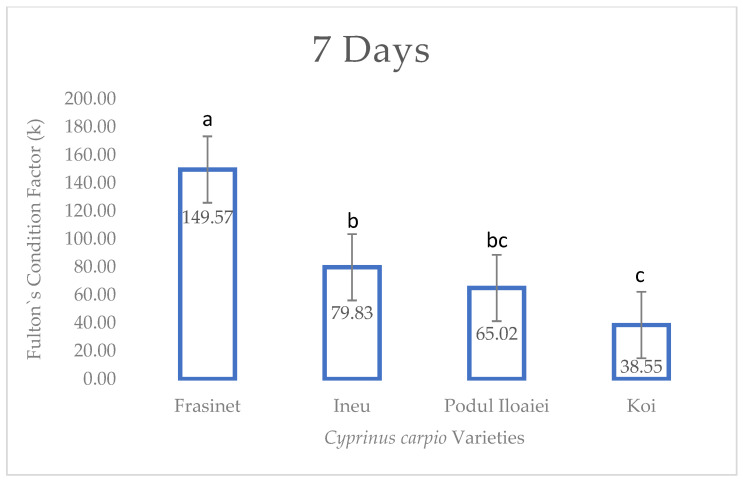
Fulton’s condition factor of *Cyprinus carpio* varieties at 7 days post-hatch. Values are presented as mean ± SEM (*n* = 30). Different superscript letters above bars indicate significant differences between varieties (Tukey’s HSD test, *p* < 0.05). F-value = 23.45; *p* < 0.001.

At 7 days post-hatch (Figure 1), significant differences were observed among the varieties (F = 23.45, *p* < 0.001). Frăsinet exhibited the highest Fulton’s index (149.57 ± 17.485a), followed by Ineu (79.83 ± 9.173b), Podu Iloaiei (65.02 ± 5.108b), and Koi (38.55 ± 5.417c).

**Figure 2:**
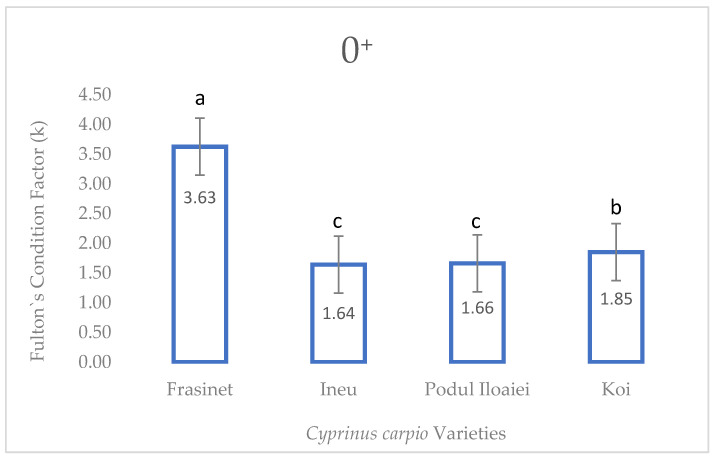
Fulton’s condition factor of *Cyprinus carpio* varieties at summer fingerling stage (0^+^). Values are presented as mean ± SEM (*n* = 30). Different superscript letters above bars indicate significant differences between varieties (Tukey’s HSD test, *p* < 0.05). F-value = 20.76; *p* < 0.001.

For the 0^+^ stage, Frăsinet maintained the highest Fulton’s index (3.63 ± 0.018a), significantly different from all other varieties (F = 20.76, *p* < 0.001). Koi showed the second-highest value (1.85 ± 0.017b), while Podu Iloaiei and Ineu had lower similar values (1.66 ± 0.014c and 1.64 ± 0.012c, respectively) (Figure 2).

**Figure 3:**
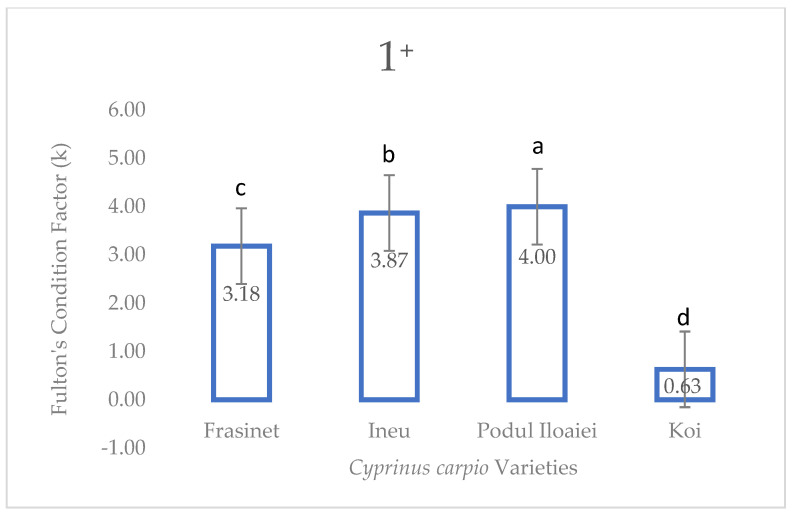
Fulton’s condition factor of *Cyprinus carpio* varieties at one year and summer stage (1^+^). Values are presented as mean ± SEM (*n* = 30). Different superscript letters above bars indicate significant differences between varieties (Tukey’s HSD test, *p* < 0.05). F-value = 24.92; *p* < 0.001.

At the 1^+^ stage, significant differences were observed among varieties (F = 24.92, *p* < 0.001). Podu Iloaiei exhibited the highest Fulton’s index (4.00 ± 0.040a), followed closely by Ineu (3.87 ± 0.024a) and Frăsinet (3.18 ± 0.014b). Koi showed a significantly lower value (0.63 ± 0.011c) compared to other varieties (Figure 3).

**Figure 4:**
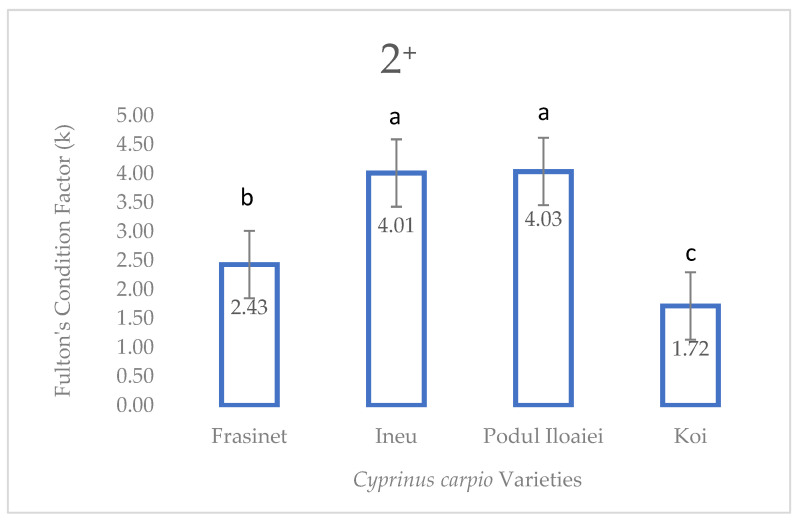
Fulton’s condition factor (K) in four varieties of *Cyprinus carpio* at age 2^+^. Values are presented as means ± standard error (*n* = 30 per variety). Different letters above bars indicate significant differences between varieties (Tukey’s HSD test, *p* < 0.001). Fulton’s condition factor was calculated as K = (W/L^3^) × 100, where W is weight in grams and L is total length in centimeters.

At the 2^+^ stage, significant differences in Fulton’s condition factor were observed among varieties (one-way ANOVA, F = 1842.95, *p* < 0.001). Podu Iloaiei and Ineu exhibited the highest Fulton’s indices (4.03 ± 0.02 and 4.01 ± 0.021, respectively), with no significant difference between them (Tukey’s post hoc test, *p* = 0.897). Frăsinet showed a significantly lower value (2.43 ± 0.012, *p* < 0.001 compared to both Podu Iloaiei and Ineu), while Koi demonstrated the lowest Fulton’s index (1.72 ± 0.044, *p* < 0.001 compared to all other varieties) (Figure 4).

These results highlight the significant variations in body shape development among different *Cyprinus carpio* varieties throughout their growth stages, which could have implications for their adaptation to different environments and their suitability for various aquaculture systems.

## 4. Discussion

The present study provides new insights into the growth dynamics and morphological development of four *Cyprinus carpio* varieties (Frăsinet, Ineu, Podu Iloaiei, and Koi) across different life stages. The findings reveal significant variations in growth performance and body shape among these varieties, which have important implications for aquaculture practices and ecological adaptations in Romania and beyond.

### 4.1. Growth Performance

The observed differences in Weight Gain (WG), Specific Growth Rate (SGR), and Relative Growth Rate (RGR) among the four varieties highlight the genetic diversity within Romanian *Cyprinus carpio* populations. The superior growth performance of Podu Iloaiei and Ineu varieties at the 1^+^ and 2^+^ stages suggests their potential for improved aquaculture productivity. These findings align with previous studies that have reported considerable variation in growth rates among different carp strains [30,31,32,33,34,35]. The results also corroborate the work of Radu [36] and Petrea [37], who observed similar growth patterns in Romanian carp varieties.

The Koi variety showed a unique growth pattern, with a lower WG at early stages but a higher SGR and RGR at the 2^+^ stage. This could be attributed to the ornamental nature of Koi, which may have been selectively bred for different traits compared to other varieties [38,39,40,41,42,43]. The lower initial growth but higher later stage growth rate in Koi warrants further investigation into the genetic and physiological mechanisms underlying this pattern [30,39,44,45,46,47], as suggested by Yue [48] in a comprehensive review of Koi carp genetics.

### 4.2. Profile Index and Morphological Development

The analysis of the profile index revealed distinct patterns of body shape development among the four Romanian varieties. The general trend of decreasing profile index with age is consistent with the allometric growth patterns typically observed in fish [49,50,51,52,53,54,55]. The variety-specific differences in this trend provide new insights into the morphological plasticity of *Cyprinus carpio* [33,56,57,58,59,60], supporting the findings of Hulata [30] on the diverse morphological traits in carp populations.

The dramatic change in profile index observed in the Podu Iloaiei variety, from the highest value at 7 days post-hatch to one of the lowest at later stages, is particularly intriguing. This rapid transformation could be an adaptive feature allowing for better survival in early life stages, followed by a more streamlined body shape for improved swimming efficiency in adults [54,55,61,62,63,64]. The maintenance of a higher profile index in Koi at the 0^+^ and 1^+^ stages aligns with the ornamental purpose of this variety, where a deeper body shape might be favored for esthetic reasons [33,38,65,66], consistent with the observations of Balon [67] on the artificial selection of Koi carp.

### 4.3. Fulton’s Condition Factor

The analysis of Fulton’s condition factor revealed significant variations among the four Romanian *Cyprinus carpio* varieties across different life stages. These differences provide insights into the overall health and energy storage of the fish, which are important factors in aquaculture and ecological contexts [53,68,69,70,71,72,73]. The findings complement the work of Gheorghe [74] on condition factors of Romanian carp strains.

The exceptionally high Fulton’s index observed in Frăsinet at 7 days post-hatch suggests a substantial energy reserve in early life stages, which could be advantageous for survival and initial growth [49,75,76,77,78], as proposed by Kamler [79] in a review of early life history in fish. The trend of increasing Fulton’s index with age in Podu Iloaiei and Ineu varieties indicates improved condition and energy storage as these varieties mature, which could be beneficial for commercial aquaculture production [53,71,72,73,80,81,82], supporting the observations of Mraz [83] on carp growth efficiency.

The Koi variety demonstrates a unique pattern, with consistently lower Fulton’s index values across all life stages. This could be attributed to the ornamental nature of Koi, where breeding selection may have focused on esthetic traits rather than condition factors [33,40,65,67,84,85]. The lower Fulton’s index in Koi suggests that this variety might require specialized nutritional management in aquaculture settings to maintain optimal health [71,86,87,88,89], as discussed by Sudirman [90] in their review of ornamental fish nutrition.

Data from previous studies (Table 5) offer points of comparison, particularly for older fish. Mirror carp at the 1^+^ stage show substantially higher weight gain compared to the 0^+^ fish in the current study, which is expected due to age difference. Various wild and selectively bred strains demonstrate a range of Fulton’s K values in adult fish, generally higher than those observed in the 0^+^ fish of the current study.

### 4.4. Implications for Aquaculture and Ecology

The varying growth patterns and morphological development observed among these Romanian *Cyprinus carpio* varieties have significant implications for aquaculture practices and ecological considerations. The superior growth performance of Podu Iloaiei and Ineu varieties at later stages suggests their potential for improved production efficiency in commercial aquaculture settings [4,5,6,7,33,40,41], aligning with the findings of Radu [92] and Bădilaș [93] on Romanian carp aquaculture. The unique growth pattern of Koi highlights the importance of considering the entire growth trajectory when selecting varieties for cultivation, as late-blooming varieties may still achieve desirable final sizes [6,33,72,73,94,95].

The differences in profile index development could influence the varieties’ adaptability to different aquaculture systems and natural habitats. Varieties with lower profile indices at adult stages, such as Ineu and Podu Iloaiei, may be better suited for high-density aquaculture systems or habitats with stronger water currents due to potentially improved swimming efficiency [75,96,97,98,99,100]. Conversely, varieties maintaining higher profile indices, like Frăsinet, might be more adapted to lentic environments or extensive aquaculture systems [30,101,102,103,104,105].

While the study presents valuable data on the variability of Romanian carp varieties and provides new insights into their growth performance and morphological development, a significant limitation is the absence of genetic and hormonal analyses, which restricts our understanding of the underlying mechanisms.

### 4.5. Future Research Directions

To further understand the growth and morphological differences in Romanian *Cyprinus carpio* varieties, future studies should focus on genetic analysis to identify growth-related QTL [40,42,45,46,48,106,107], coupled with physiological studies on metabolic differences, especially in Koi [108,109,110,111,112,113,114,115]. Additionally, environmental interaction studies under various rearing conditions [30,33,116,117,118,119,120,121] and long-term performance evaluations in different aquaculture systems [4,25,31,33,122,123,124] are important. These research directions will enhance our understanding of Romanian carp varieties, benefiting both local aquaculture and global carp breeding research.

### 4.6. Limitations in Genetic Interpretation and Alternative Explanations

While our study demonstrates clear phenotypic differences among carp varieties, we must carefully consider alternative explanations for the observed variations. The superior growth performance of Podu Iloaiei and Ineu varieties (WG: 849.73 ± 4.09 g and 846.25 ± 5.17 g at 2^+^ stage, respectively) could result from multiple factors beyond genetic diversity, as phenotypic expression in carp is influenced by complex interactions between genetic and environmental factors [30].

The observed variations might primarily reflect phenotypic plasticity rather than fixed genetic differences. Significant plasticity in carp development has been well documented, with early-life environmental conditions substantially influencing adult morphology and growth patterns [67]. The dramatic change in Podu Iloaiei’s profile index (from 4.22 at 7 days to 2.18 at 2^+^) could represent such plastic responses to environmental conditions rather than genetic predetermination. This interpretation is further supported by the variety’s adaptive responses across different developmental stages.

Environmental factors present another significant consideration in interpreting our results. Although water quality parameters were maintained within optimal ranges at both facilities, subtle differences in pond ecosystems, including natural productivity, substrate composition, and microbial communities, could significantly influence growth patterns. Environmental variations can substantially affect carp growth and condition factors, suggesting that observed differences might reflect local ecological conditions rather than inherent genetic variation [71].

Maternal effects represent another crucial factor in interpreting early growth patterns. Maternal effects can persist through several growth stages in carp, suggesting that the superior early growth observed in some varieties might reflect maternal investment rather than genetic superiority [33]. This consideration is particularly relevant when evaluating early-life performance differences among varieties.

Historical selection pressures must also be considered in interpreting growth patterns. Local adaptation in carp can result from both intentional selection and environmental pressures over multiple generations [32]. Without direct genetic data, the observed growth patterns might reflect historical selection events rather than current genetic diversity, complicating the interpretation of variety-specific traits.

These limitations and alternative explanations suggest several critical directions for future research. Controlled common-garden experiments would be essential to separate genetics from environmental effects, while a molecular genetic analysis could quantify actual genetic diversity among varieties. Multi-generational studies would be valuable in assessing the stability of observed traits, and an investigation of epigenetic mechanisms could reveal how environmental factors influence growth patterns across generations.

This more nuanced interpretation cautions against oversimplified genetic explanations for phenotypic variation in carp populations [57]. While our results provide valuable insights for aquaculture practices, they should be viewed as preliminary evidence requiring further genetic investigation rather than definitive proof of genetic differentiation among varieties. Future studies integrating both genetic and environmental analyses would provide a more comprehensive understanding of variety-specific traits in Romanian carp populations.

### 4.7. Management Strategies for Koi Production

The unique growth pattern observed in Koi, characterized by lower initial Weight Gain but higher late-stage Specific Growth Rate (0.18 ± 0.00% day^−1^) and Relative Growth Rate (0.98 ± 0.05 g day^−1^), necessitates specific management strategies to optimize production. Our findings suggest a comprehensive approach to Koi cultivation that adapts to the variety’s developmental stages and ornamental requirements.

Stocking density management emerges as a critical factor in optimizing Koi production. During the larval to 0^+^ stage, higher densities of 100–150 larvae/m^2^ are feasible due to the fish’s smaller size and relatively uniform growth patterns [113]. As development progresses to the 1^+^ stage, density should be reduced to 20–25 fish/m^2^ to accommodate increasing growth variation and individual development. A further reduction to 10–15 fish/m^2^ during the 2^+^ stage optimizes the late-stage growth potential and allows for enhanced color development [114].

Nutritional management must be tailored to support both growth performance and ornamental quality. Early developmental stages (0^+^) require higher protein content (38–42%) to support rapid growth and physiological development. The 1^+^ stage benefits from balanced protein–lipid ratios (35% protein, 8% lipids), supporting both growth and color enhancement. During the 2^+^ stage, when Koi exhibit accelerated growth rates, increased feeding frequency (3–4 times daily) effectively capitalizes on the enhanced growth potential while maintaining esthetic qualities [89].

Production cycle optimization requires careful consideration of both biological and market factors. The extended cultivation period through the 2^+^ stage maximizes growth potential and allows for full color development. Implementation of size grading at the 1^+^ stage optimizes population management and reduces competition, while the synchronization of harvest with peak market demand periods enhances economic returns. Environmental management plays a crucial role, particularly during periods of accelerated growth. Maintaining optimal water quality parameters, providing adequate shelter structures, and implementing partial water exchange during the 2^+^ stage supports increased metabolic demands while minimizing stress.

These management recommendations are particularly important given Koi’s value as an ornamental variety, where both growth and esthetic qualities must be considered [90]. The late-stage growth acceleration can be advantageously utilized through proper feeding and density management strategies while maintaining conditions that preserve ornamental quality. This integrated approach to Koi production acknowledges both the variety’s unique growth patterns and its primary market as ornamental fish, offering a balanced strategy for optimizing both growth performance and esthetic value. Implementation of these recommendations should be calibrated according to specific facility conditions and market requirements, ensuring optimal outcomes in various production contexts.

### 4.8. Evolutionary Context and Management Implications

The patterns observed in this study reflect complex interactions between evolutionary history, artificial selection, and environmental adaptation. The diversity among Romanian carp varieties manifests through distinct morphological and physiological traits that likely result from both intentional breeding programs and local adaptation to specific environmental conditions over multiple generations.

The evolutionary and ecological implications of these findings are substantial. Podu Iloaiei’s superior growth performance (WG: 849.73 ± 4.09 g at 2^+^) combined with the high Fulton’s condition factor (4.03 ± 0.02) suggests adaptation to productive lowland pond ecosystems, while Ineu’s efficient growth pattern (WG: 846.25 ± 5.17 g) and streamlined body shape (profile index: 2.16 ± 0.004) indicates optimization for more dynamic water conditions. Frăsinet’s distinctive early-life characteristics, particularly its high initial Fulton’s index (149.57 ± 17.485), point to selection pressures favoring early-life survival. In contrast, Koi’s unique growth trajectory emphasizes the impact of artificial selection for ornamental traits over production characteristics.

The observed phenotypic plasticity, particularly in profile indices and stage-specific growth patterns, likely represents adaptive responses to environmental variability. These plastic responses may serve as buffering mechanisms against environmental uncertainties, suggesting evolved life history strategies specific to each variety. This interpretation aligns with the recent understanding of phenotypic plasticity as an adaptation mechanism in fish populations [67,71].

Based on these evolutionary and developmental patterns, variety-specific management strategies emerge as important for optimizing production. Podu Iloaiei’s characteristics suggest optimal performance in semi-intensive pond systems with moderate stocking densities (15–20,000 fish/ha) and standard protein feeding regimes (32% protein). Ineu’s morphological and physiological traits indicate suitability for flow-through systems with higher oxygen requirements (>6 mg/L) and increased stocking densities (20–25,000 fish/ha). Frăsinet’s distinct early-life characteristics necessitate enhanced nutritional management during initial development stages, with emphasis on protein content (35%) and structured habitat provisions.

Koi production requires a specialized approach acknowledging its unique growth pattern and ornamental value. The variety’s late-stage growth acceleration (SGR: 0.18 ± 0.00% day^−1^) suggests benefits from extended cultivation periods with careful attention to environmental parameters (23–25 °C) and specialized feeding regimes aligned with color development stages. This management approach must balance growth optimization with the preservation of ornamental qualities.

These management strategies should be contextualized within local environmental conditions, market requirements, and available infrastructure. The distinct growth patterns and morphological characteristics of each variety reflect their evolutionary history and potential for aquaculture optimization. This diversity represents a valuable genetic resource for Romanian aquaculture, offering multiple options for different production systems and market requirements.

Understanding the evolutionary basis of variety-specific traits enables more effective management strategies. Future developments should focus on integrating traditional knowledge with modern aquaculture practices while maintaining genetic diversity and addressing market demands. This approach acknowledges both the evolutionary background and practical aquaculture requirements, providing a framework for optimized production while preserving the unique characteristics of each variety.

## 5. Conclusions

This investigation of Romanian *Cyprinus carpio* varieties reveals distinctive developmental trajectories with significant implications for aquaculture optimization. The remarkable performance of Podu Iloaiei (WG: 849.73 ± 4.09 g, Fulton’s factor: 4.03 ± 0.02 at 2^+^ stage) establishes its potential for intensive commercial aquaculture, while Ineu’s streamlined morphology (profile index: 2.16 ± 0.004) suggests advantages in flow-through systems. Frăsinet’s high early-life condition factor and Koi’s unique late-stage growth patterns (SGR: 0.18 ± 0.00% day^−1^) demonstrate the importance of variety-specific management strategies.

These findings provide a foundation for developing targeted breeding programs and optimizing production protocols while emphasizing the importance of preserving local varieties as genetic resources. Future research should focus on elucidating the genetic basis of these growth patterns to enhance breeding programs and cultivation strategies, ultimately contributing to aquaculture sustainability and productivity.

## Figures and Tables

**Table 1:** Weight Gain (WG) of *Cyprinus carpio* varieties in different ontogenic stages.

*Cyprinus carpio* Variety	WG0^+^ (g)	WG1^+^ (g)	WG2^+^ (g)
Frasinet	37.55 ± 0.15a	471.13 ± 1.78c	834.45 ± 5.28a
Ineu	36.27 ± 0.11c	560.79 ± 2b	846.25 ± 5.17a
Podul Iloaiei	37.37 ± 0.14a	583.65 ± 1.68a	849.73 ± 4.09a
Koi	36.75 ± 0.08b	77.73 ± 2.1d	403.99 ± 14.21b
F-value	12.45	24.67	18.92
*p*-value	<0.001	<0.001	<0.001

Values are presented as mean ± SEM (*n* = 30). Different letters (a, b, c, d) within columns indicate significant differences between varieties (Tukey’s HSD test, *p* < 0.05). WG = Weight Gain, calculated as Final Weight−Initial Weight. WG0^+^ = Weight Gain at summer fingerling stage; WG1^+^ = Weight Gain at one year and summer stage; WG2^+^ = Weight Gain at two years and summer stage.

**Table 2:** Specific Growth Rate (SGR) of *Cyprinus carpio* varieties in different ontogenic stages.

*Cyprinus carpio* Variety	SGR0^+^	SGR1^+^	SGR2^+^
Frasinet	2.2 ± 0.05b	0.31 ± 0b	0.12 ± 0b
Ineu	2.57 ± 0.07a	0.33 ± 0a	0.1 ± 0c
Podul Iloaiei	2.73 ± 0.04a	0.33 ± 0a	0.1 ± 0c
Koi	2.7 ± 0.09a	0.13 ± 0c	0.18 ± 0a
F-value	15.32	21.84	19.76
*p*-value	<0.001	<0.001	<0.001

Values are presented as mean ± SEM (*n* = 30). Different letters (a, b, c) within columns indicate significant differences between varieties (Tukey’s HSD test, *p* < 0.05). SGR = Specific Growth Rate, calculated as [(ln Final Weight − ln Initial Weight)/time (days)] × 100. SGR0^+^ = Specific Growth Rate at summer fingerling stage; SGR1^+^ = Specific Growth Rate at one year and summer stage; SGR2^+^ = Specific Growth Rate at two years and summer stage.

**Table 3:** Relative Growth Rate (RGR) of *Cyprinus carpio* varieties in different ontogenic stages.

*Cyprinus carpio* Variety	RGR0^+^	RGR1^+^	RGR2^+^
Frasinet	119.98 ± 21.13b	3.4 ± 0.02b	0.45 ± 0b
Ineu	351.31 ± 122.65ab	4.21 ± 0.02a	0.39 ± 0b
Podul Iloaiei	322.27 ± 33.76ab	4.26 ± 0.02a	0.38 ± 0b
Koi	508.59 ± 141.95a	0.58 ± 0.02c	0.98 ± 0.05a
F-value	3.27	25.91	17.83
*p*-value	0.045	<0.001	<0.001

Values are presented as mean ± SEM (*n* = 30). Different letters (a, b, c) within columns indicate significant differences between varieties (Tukey’s HSD test, *p* < 0.05). RGR = Relative Growth Rate, calculated as [(Final Weight − Initial Weight)/Initial Weight] × 100. RGR0^+^ = Relative Growth Rate at summer fingerling stage; RGR1^+^ = Relative Growth Rate at one year and summer stage; RGR2^+^ = Relative Growth Rate at two years and summer stage.

**Table 4:** Profile index of *Cyprinus carpio* varieties in different ontogenic stages.

*Cyprinus carpio* Variety	7 Days	0^+^	1^+^	2^+^
Frasinet	3.06 ± 0.07c	2.57 ± 0.006b	2.4 ± 0.005b	2.43 ± 0.005a
Ineu	2.9 ± 0.07c	2.44 ± 0.009c	2.17 ± 0.005c	2.16 ± 0.004b
Podul Iloaiei	4.22 ± 0.149a	2.45 ± 0.009c	2.18 ± 0.008c	2.18 ± 0.004b
Koi	3.69 ± 0.101b	2.93 ± 0.023a	2.48 ± 0.005a	2.17 ± 0.005b
F-value	28.64	19.82	22.15	16.93
*p*-value	<0.001	<0.001	<0.001	<0.001

Values are presented as mean ± SEM (*n* = 30). Different letters (a, b, c) within columns indicate significant differences between varieties (Tukey’s HSD test, *p* < 0.05). Profile index calculated as Maximum Height/Total Length. Measurements taken at 7 days post-hatch, summer fingerling stage (0^+^), one year and summer stage (1^+^), and two years and summer stage (2^+^).

**Table 5:** Comparative growth performance and morphological data from various *Cyprinus carpio* studies.

Study	Variety/Strain	Age	Weight Gain (g)	SGR (%/Day)	Fulton’s K	Profile Index
Current Study	Frăsinet	1^+^	471.13 ± 1.78	0.31 ± 0	3.18 ± 0.014	2.4 ± 0.005
	Ineu	1^+^	560.79 ± 2	0.33 ± 0	3.87 ± 0.024	2.17 ± 0.005
	Podu Iloaiei	1^+^	583.65 ± 1.68	0.33 ± 0	4 ± 0.04	2.18 ± 0.008
	Koi	1^+^	77.73 ± 2.1	0.13 ± 0	0.63 ± 0.011	2.48 ± 0.005
[28]	Mirror carp	1^+^	583.7 ± 12.3	1.69 ± 0.01	3.24 ± 0.02	-
[29]	Various strains	1^+^	329–438	1.51–1.66	-	-
[91]	Various strains	1^+^	441–507	-	-	-
[33]	Wild carp	Adult	-	-	1.69–2.15	-
[35]	Various strains	Adult	-	-	1.72–2.86	-
[36]	Selective bred	1^+^	539.8 ± 149.2	-	-	-
[66]	Wild carp	Adult	-	-	1.60–1.78	-

## Data Availability

All data generated during this study are present in this article.
